# Advanced Energy Management Strategy of Photovoltaic/PEMFC/Lithium-Ion Batteries/Supercapacitors Hybrid Renewable Power System Using White Shark Optimizer

**DOI:** 10.3390/s23031534

**Published:** 2023-01-30

**Authors:** Hesham Alhumade, Hegazy Rezk, Mohamed Louzazni, Iqbal Ahmed Moujdin, Saad Al-Shahrani

**Affiliations:** 1 Chemical and Materials Engineering Department, Faculty of Engineering, King Abdulaziz University, Jeddah 21589, Saudi Arabia; 2 Center of Excellence in Desalination Technology, King Abdulaziz University, Jeddah 21589, Saudi Arabia; 3 K. A. CARE Energy Research and Innovation Center, King Abdulaziz University, Jeddah 21589, Saudi Arabia; 4 Department of Electrical Engineering, College of Engineering in Wadi Alddawasir, Prince Sattam bin Abdulaziz University, Wadi Alddawasir 11991, Saudi Arabia; 5 Department of Electrical Engineering, Faculty of Engineering, Minia University, Minia 61519, Egypt; 6 Science Engineer Laboratory for Energy, National School of Applied Sciences, Chouaib Doukkali University of El Jadida, El Jadida 24000, Morocco; 7 Department of Mechanical Engineering, King Abdulaziz University, Jeddah 21589, Saudi Arabia

**Keywords:** energy management systems, hydrogen fuel, renewable energy, microgrid, optimization

## Abstract

The slow dynamic response of a proton exchange membrane fuel cell (PEMFC) to high load change during deficit periods must be considered. Therefore, integrating the hybrid system with energy storage devices like battery storage and/or a supercapacitor is necessary. To reduce the consumed hydrogen, an energy management strategy (EMS) based on the white shark optimizer (WSO) for photovoltaic/PEMFC/lithium-ion batteries/supercapacitors microgrid has been developed. The EMSs distribute the load demand among the photovoltaic, PEMFC, lithium-ion batteries, and supercapacitors. The design of EMSs must be such that it minimizes the use of hydrogen while simultaneously ensuring that each energy source performs inside its own parameters. The recommended EMS-based-WSO was evaluated in regard to other EMSs regarding hydrogen fuel consumption and effectiveness. The considered EMSs are state machine control strategy (SMCS), classical external energy maximization strategy (EEMS), and optimized EEMS-based particle swarm optimization (PSO). Thanks to the proposed EEMS-based WSO, hydrogen utilization has been reduced by 34.17%, 29.47%, and 2.1%, respectively, compared with SMCS, EEMS, and PSO. In addition, the efficiency increased by 6.05%, 9.5%, and 0.33%, respectively, compared with SMCS, EEMS, and PSO.

## 1. Introduction

Microgrids offer a potential solution for integrating small-scale renewable energy sources and loads along with energy storage systems and other non-renewable sources [1]. Many issues, including finance and cost concerns, are debated in multiple international seminars and meetings. The main topics of these seminars are innovative efforts and funding feasibility. The development of several wireless and IoT technologies improved the performance of microgrids [2,3]. Manufacturers today are also using microgrids’ capabilities, mainly to lessen their dependency on long-distance distribution lines and, consequently, to cut down on transmission losses [4]. The main benefits and challenges of the microgrid are reported in Table 1.

A microgrid can be customized to meet each customer’s individual needs by mixing various components, offering the best technical and financial options [5]. These systems meet energy demands for electrical and/or thermal energy typically supplied by the natural gas or electric utility company. Microgrid components can sometimes be found in the following list [6]:

○ Distributed energy resources (DERs): include traditional generators like Gensets or natural gas generators that mechanically transform fuel into electricity and heat, as well as renewable energy technologies, including solar and wind power, which operate on natural renewable resources.
○ Energy storage systems (ESSs): electrochemical (batteries), mechanical (flywheels), and thermal (hot water) systems are examples of systems. This energy can originate from renewable overproduction or be charged when energy is cheaper for peak periods.
○ Loads: the user loads can be classified as industrial, residential, commercial, and electric vehicles (EVs). In addition, the loads can be classified according to their nature as crucial and non-crucial loads or controllable and non-controllable loads.
○ Monitoring and Control systems: monitoring systems are required in data acquisition and transmission, whereas intelligent controls are implemented to improve electrical efficiency and cost performance by automatically directing supply to the most efficient available source.

Modularity, scalability, energy management, and resource balancing are essential characteristics of a successful microgrid. The DERs can deliver high performance regardless of the site’s conditions using a proper energy management strategy, whether off-grid or on-grid [7].

PEMFC transforms chemical reactions to DC power. It earned great consideration thanks to its simple size, quietness, and fewer effects on the atmosphere. Several applications, such as trains, aircraft, and electric vehicles, have used PEMFC in recent years [8]. However, the PEMFC has restrictions on insufficient power density and postponed dynamic response. Therefore, the installation of either battery storage (BSs) or supercapacitors (SCs) to build a hybrid system may improve system performance [9,10]. It is critical to have an energy-management plan into a hybrid-based system that combines storage systems as well as photovoltaic (PV) and wind turbines are examples of sources of clean energy (WT) and PEMFCs (EMSs) [10]. The remaining energy here between diverse sources is controlled by the EMSs. Plus, they facilitate proper production and lengthen the authentication system.

In the literature, the EMS can be classified into three major categories: rule-based, artificial intelligence, and optimization-based strategies [11,12]. Rule-based strategies can be classified into two subcategories: deterministic and Boolean logic strategies. Furthermore, artificial intelligence can be classified into three subcategories: fuzzy logic, neural network, and Machin learning strategies. Finally, optimization-based strategies, which aim to minimize/maximize the objective function. The objective function might include emissions, costs, energy savings, device deterioration, and global efficiency [13]. There are two subcategories for optimization-based strategies: online and offline. The offline optimization, including dynamic programming (DP) [14,15], nonlinear programming (NLD) [16], stochastic dynamic control strategy (SDP) [17], and genetic algorithm (GA) [18], are the most widely utilized to manage the power flow in the DCMG. The existence of all load profiles is required. Hence, a large amount of data is involved. This makes their implementation extremely difficult. Whereas in the online optimization, the objective function depends only on the actual states of the system [19]. They have more restricted processing and higher real-time performance. The SoC of the storage devices is taken into account, but global optimization is not possible. The equivalent consumption minimization strategy (ECMS), model predictive control (MPC) [20], and the external energy maximization strategy (EEMS) are examples of these techniques.

The main contribution of this work is proposing, for the first time, an efficient EMS based on white shark optimizer (WSO) for photovoltaic/PEMFC/lithium-ion batteries/supercapacitors (PV/PEMFC/BSs/SCs) microgrid. The two critical aspects are minimizing hydrogen consumption and improving the hybrids’ system performance. To prove the superiority of the proposed strategy, three EMSs are considered: state machine control strategy (SMCS), classical methodology for maximizing external power supply (EEMS), and optimized EEMS-based particle swarm optimization (PSO).

The description of the different components of the hybrid PV/PEMFC/BSs/SCs system is presented in Section 2. Section 3 explains considered energy management strategies. The results are presented and discussed in detail in Section 4. Finally, the main findings are concluded in Section 5.

## 2. Modeling of the PV/PEMFC/BSs/SCs Hybrid System

This part deals with the description and modeling of the hybrid renewable energy system component from a photovoltaic array, fuel cell, battery storage, and supercapacitor. The photovoltaic array generates and injects variable energy into the load under different meteorological conditions. Due to its low electricity production, SCs are employed to maintain and absorb oscillating power struggles as well as transient/fluctuating system changes. In addition to having a very large number of cycles with excellent results and no deterioration, SCs are efficient in extremely quick charging and discharges [21]. The battery is used to store the electricity produced by the solar generator so that it may be accessed at a later point in time, if necessary. However, because of its high energy density, it seems to sustain continual power adjustments and only provide temporary power in urgent situations.

### 2.1. Modeling of PV Array

**Figure 1:**
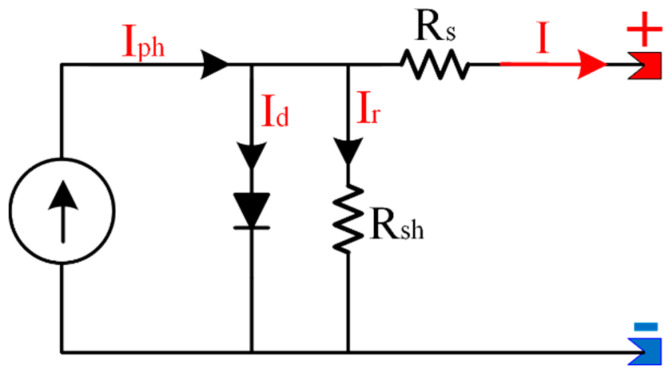
Equivalent circuit of the solar cell model.

A single-diode electrical model is shown in Figure 1. The electrical model consists of a photocurrent parallel to a diode, a shunt resistance, and a series resistance. Based on KVL, the equations that control this circuit is presented as [22]:

(1) $$\left\{ \begin{array}{l} I=I_{ph}-I_{d}-I_{r}\\ I=I_{ph}-I_{0}\left[e^{\frac{V+I\cdot R_{s}}{nV_{th}}}-1\right]-\frac{V+I\cdot R_{s}}{R_{sh}} \end{array}\right.$$

*R_sh_* and *R_s_* represent shunt and series resistances, respectively. *n* is the ideality factor, *I*_0_ is the saturation current, and *V_th_* is the thermal voltage given by the following relation:

(2) $$V_{th}=\frac{k\cdot T_{c}}{q}$$

where *q* seems to be the electron’s charge, *T_c_* is the temperature of the cell, and *k* is the Boltzmann constant.

The photocurrent is represented as:

(3) $$I_{ph}=\left[I_{SC}-\alpha \left(T_{c}-T_{r}\right)\right]G$$

where *Tc* and *T_r_* are the experimental and benchmark temperatures, *α* is the temperature coefficient, and *G* is the solar radiation. *I_SC_* is the short circuit current in A. In sum, considering series and parallel panels, the current produced by the PV array can be estimated as follows:

(4) $$I=I_{ph}-I_{0}\left[e^{\frac{V_{out}+I_{out}\cdot N_{S}\cdot R_{S}}{n\cdot N_{S}\cdot V_{th}}}-1\right]-\frac{V_{out}+I_{out}\cdot N_{S}\cdot R_{S}}{R_{sh}\cdot N_{S}}$$

where *V_out_* is the voltage and *I_out_* is the current of the photovoltaic array, respectively. *N_S_* and *N_P_* are the number of series and parallel cells connected in photovoltaic arrays.

### 2.2. Battery Storage Model

**Figure 2:**
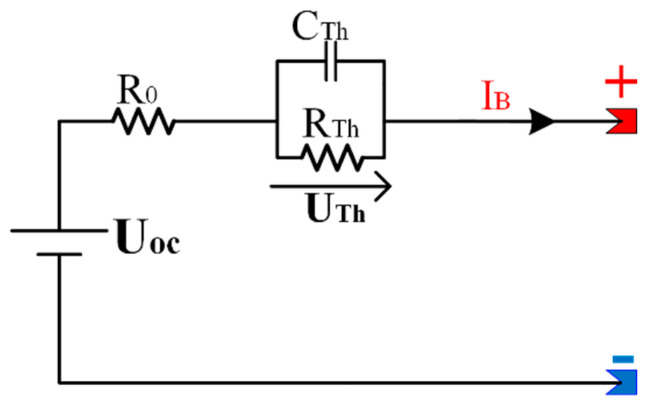
Equivalent circuit of Li-ion battery Model.

In industry, there are different types of batteries. The model adopted in the current case analysis is a lithium-ion relying on the Thevenin representation [23], which is demonstrated in Figure 2. Since lithium-ion batteries have a large storage capacity, a high proportion of operating voltage, and a prolonged cycle life, they are frequently employed due to their benefits over other energy storage technologies. The presented electrical model is among the most popular energy storage technologies. It contains an internal resistance with a parallel RC network. The open-circuit voltage *U_oc_*, intrinsic resistances, and analogous capacitor values are the three basic elements that make up the model [24]. The intrinsic impedances are made up of the polarization resistance *R_Th_* and the conductive resistance *R*_0_. The battery’s charging and discharging dynamics are represented by the roughly comparable capacitance *C_Th_*. *U_Th_* denotes the voltage above *C_Th_*.

The following equation is a characterization of the supply voltage:

(5) $$\left\{ \begin{array}{l} U_{B}=U_{oc}-R_{0}\cdot I_{B}-U_{Th}\\ {\dot{U}}_{Th}=-\frac{U_{Th}}{R_{Th}\cdot C_{Th}}+\frac{I_{B}}{C_{Th}} \end{array}\right.$$

### 2.3. PEMFC Mathematical Model

**Figure 3:**
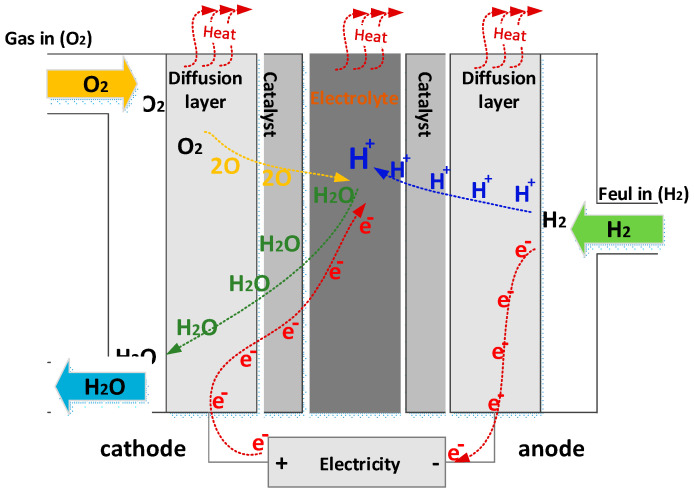
Structure of PEMFC.

As shown in Figure 3 [25], a fuel cell includes an anode and a cathode, as well as an electrolyte. At the anode, a platinum catalyst splits the hydrogen atom into protons and electrons. While the electrons receive current from an externally applied to produce the FC output voltage, the protons reach the cathode. At the cathode, the protons and electrons are once again coupled with oxygen to produce heat and water.

The following is a presentation of the FC chemical reactions [25]:

(6) $$\left\{ \begin{array}{l} H_{2}\to 2H^{+}+2e^{-}\\ O_{2}+4e^{-}\to 2O^{-2}\\ H_{2}+\frac{1}{2}O_{2}\to H_{2}O+electrical\begin{array}{c} energy \end{array}+heat \end{array}\right.$$

**Figure 4:**
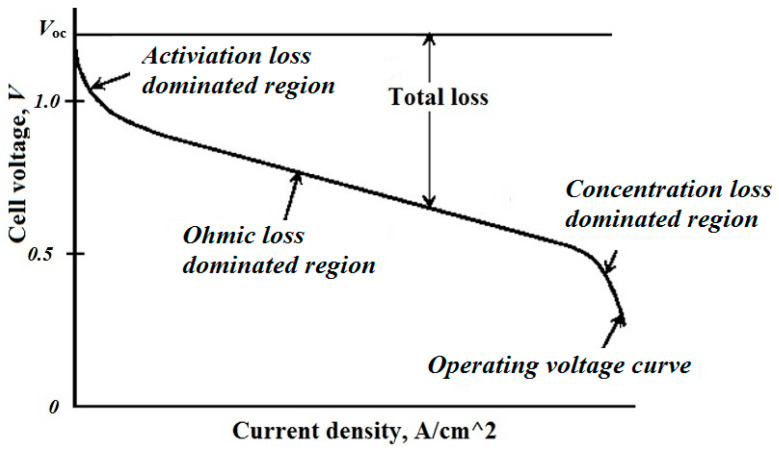
Voltage against current density characteristics.

Inside the PEMFC, there are three different types of losses: activation, ohmic, and concentration losses. Figure 4 depicts the fluctuation of PEMFC voltage with cell current density [26].

As a result, the output voltage of the PEMFC is the sum of four voltage provided by thermodynamic effect as *E_Nernst_*, the activation voltage losses zone creating the *V_act_*, the ohmic voltage losses *V_ohm_* come from the ohmic loss dominated region due to the ionic and electronic resistance, the last region generated the concentration voltage losses, *V_con_,* due to the mass transport, and *n_cell_* percent is the number of connected cells in PEMFC. In principle, the true voltage output of a fuel cell may be calculated by initiating with both the thermally anticipated voltage and removing the different voltage fluctuation inefficiencies, as shown below:

(7) $$V_{FC}=n_{cell}\left(E_{Nernest}-V_{con}-V_{act}-V_{ohm}\right)$$

Each element in Equation (1) is recognized and analyzed separately as follows:

(8) $$E_{Nernest}=E_{0}-0.85\times 10^{-3}\cdot \left(T-298.15\right)+4.3085\times 10^{-5}\cdot T\cdot \ln\left(P_{H2}\sqrt{P_{O2}}\right)$$

where *T* represents the cell temperature, and *E*_0_ = 1.229 is the thermodynamic voltage of a conventional bidirectional FC. *P_H_*_2_ and *P_O_*_2_ are the partial pressures of hydrogen and oxygen, respectively.

The reactant concentration variations at the electrode surface cause a voltage decrease in accumulation, which is characterized as:

(9) Vcon=−b·ln(1−IAImax)

where *b* represents the concentrations degradation constant and *I*_max_ represents the rated current destiny.

The activation voltage loss is determined as follows [27]:

(10) $$V_{act}=-\left[\xi_{1}+\xi_{2}T+\xi_{3}T\ln\left(C_{O2}\right)+\xi_{4}T\ln\left(I\right)\right]$$

where *ξ_i_* (*i* = 1, 2, 3, 4) symbolizes the semi-empirical parameters of the FC derived from the fluid mechanic, thermodynamic, and electrochemistry, and *C_O_*_2_ illustrates the oxygen concentration,

(11) $$C_{O2}=\frac{P_{O2}}{5.08\times 10^{6}\cdot \exp\left(\frac{-498}{T}\right)}$$

*R_M_* stands for identical membrane resistance to proton conduction, and *ρ_M_* and *l* are both the thickness of the membrane, and *λ* presents membrane water content.

(12) $$\left\{ \begin{array}{l} R_{M}=\frac{\rho_{M}\cdot l}{A}\\ {}_{M}=\frac{181.6\cdot \left[1+0.03\cdot \left(\frac{i}{A}\right)+0.062\cdot \left(\frac{T}{303}\right)\cdot {\left(\frac{i}{A}\right)}^{2.5}\right]}{\left[\lambda -0.634-3\cdot \left(\frac{i}{A}\right)\right]\cdot \exp\left[4.18\cdot \left(\frac{T-303}{T}\right)\right]} \end{array}\right.$$

The equivalent resistance of the FC causes the ohmic voltage loss, and it can be expressed as:

(13) $$V_{ohm}=I\cdot \left(R_{M}+R_{C}\right)$$

where *R_c_* is the equivalent resistance representing the concentration processes.

The stack terminal voltage of the FC is given by the number of fuel cells interconnected in series:

(14) $$V_{stack}=n\cdot V_{FC}=n\cdot \left(E_{Nernest}-V_{act}-V_{ohm}-V_{con}\right)$$

### 2.4. Supercapacitor

**Figure 5:**
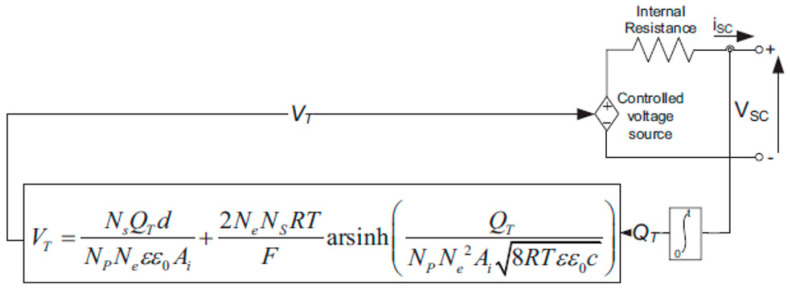
Supercapacitor equivalent circuit model.

The capacitance used in the SC model represents how the SC performs when being charged and discharged. A series resistance is analogous to those same resistances for both charging and discharging [28]. Figure 5 depicts the related system model of SC. The behavior of the SC is represented by the Stern model, which is available in the MATLAB/Simulink [29].

Once resistive losses are taken into account, the SC output voltage may be represented as:

(15) $$V_{SC}=\frac{Q_{T}}{C_{T}}-R_{SC}\cdot \;i_{SC}\;$$

with,

(16) $$\;\left\{ \begin{array}{l} C_{T}=\frac{N_{p}}{N_{s}}\cdot \;C\\ Q_{T}=N_{p}\cdot \;Q_{c}=\int i_{SC}dt\\ C={\left[\frac{1}{C_{H}}+\frac{1}{C_{GC}}\right]}^{-1}\\ C_{H}=\frac{N_{e}{\varepsilon \varepsilon }_{0}A_{i}}{d}\\ C_{GC}=\frac{FQ_{c}}{2N_{e}RT}\;\sin h\left(\frac{Q_{c}}{N_{e}{}^{2}A_{i}\sqrt{8RT{\varepsilon \varepsilon }_{0}}c}\right) \end{array}\right.$$

where *C_T_* denotes the sum of the capacitances (F), *Q_T_* represents the entire amount of electricity charged, *R_SC_* denotes intrinsic resistance (ohm), *i_SC_* is the SC current (A), *N_S_* is the number in series, *N_P_* denotes the number in parallel, *C_H_* is the Helmholtz capacitance (F), *C_GC_* represents the capacitance of Gouy-Chapman (F), Ne is the total number of layers of the electrodes, *ε* and *ε*_0_ are the permittivity values of the electrolyte material and empty area, *A_i_* is the space between both the electrodes and the electrolyte (m^2^), *d* is the length of the Helmholtz layer (m), *Q_c_* would be the electric charge of the cell, *C* is just the molar concentration (mol m^−3^).

### 2.5. Specifications of the Hybrid System

**Figure 6:**
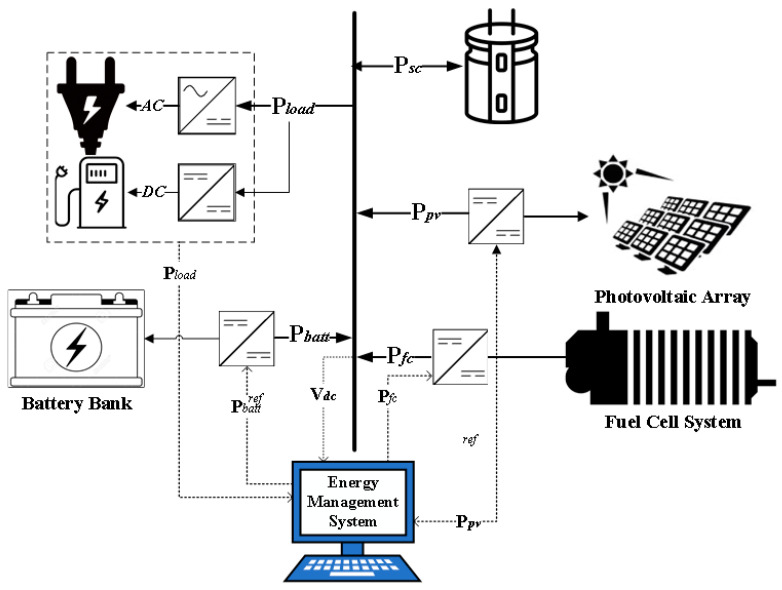
Configuration of hybrid PV/PEMFC/BSs/SCs system.

The considered hybrid power system is shown in Figure 6. It includes a PV system, PEMFC, BSs, and an SC to supply the load demand regarding high fluctuations. The perturb and observed maximum power point tracking maximizes the PV power. The specifications of the hybrid system are presented in Table 2.

## 3. Proposed Energy Management Strategies

The system’s efficiency depends greatly on the development of MG energy management, which is essential [30,31]. The necessary power by the load in a hybrid system not supplied by the PV array is known as net power (ΔP). To provide conversion efficiency, the EMS instructions might control the PEMFC, BSs, and SCs.

(17) $$\Delta P=P_{Load}-P_{PV}$$

To prevent reactive depletion, however, the FC dynamics must be controlled. In the case study, numerous EMSs are considered, including external energy optimization methodologies, state engine control, standard EEMS, and improved EMMS-based WSO.

### 3.1. Equivalent Consumption Minimization Methodology

The ECMS aims to minimize fuel consumption while maintaining a battery SOC within acceptable bounds. The accuracy of the empirical assessment of the associated fuel economy determines how successfully the ECMS performs, as stated in [32]. The related objective function is presented as follows:

(18) $$P_{Batt}^{opt}=\min(C_{FC}+\beta \cdot C_{Batt})$$

(19) $$\beta =1-2\cdot \mu \frac{SoC-0.5\left(SoC_{\min}+SoC_{\max}\right)}{SoC_{\min}+SoC_{\max}}$$

wherein *C_Bat_* denotes the battery energy usage in relation to the given energy and *μ* is a constant (0.6). This equation can be defined as follows:

(20) $$\begin{array}{c} SoC_{\min}\leq SoC\leq SoC_{\max}\\ V_{bus}^{\min}\leq V_{bus}\leq V_{bus}^{\max}\\ P_{FC}^{\min}\leq P_{FC}\leq P_{FC}^{\max} \end{array}$$

wherein *μ* is indeed a constant (0.6), and *C_Bat_* reflects the battery fuel efficiency in terms of the energy supplied.

The following equation is used to compute the FC output:

(21) $$P_{FC}^{ref}=\Delta P-P_{Grid}^{ref}-P_{Batt}^{opt}$$

**Figure 7:**
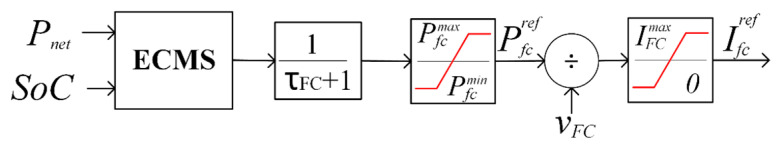
Layout of ECMS.

Figure 7 presents an illustration of the ECMS system.

### 3.2. State Machine Control Management

**Figure 8:**
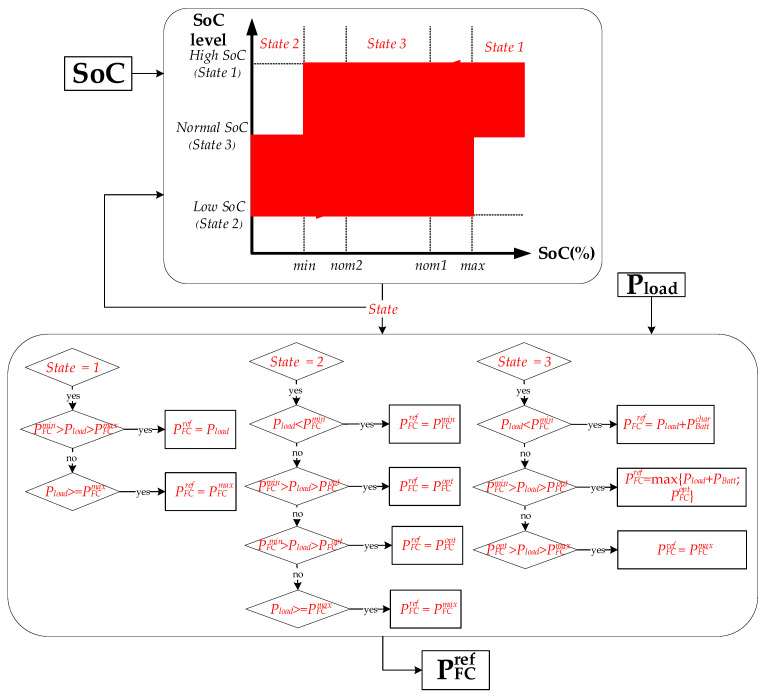
The scheme of SMC control.

An EMS depending on switching rules is designated SMC [33]. In SMC, the operational state is chosen in proportion to the inputs: ΔP and battery SOC. The battery is charged or discharged depending on the status of its inputs, as shown in Figure 8.

The numerical values of parameters addressed in SMCS are:

min = 30%; nom2 = 40%; nom1 = 85%; max = 90%;
Pfcmin = 850; Pfcmax = 8800; Pfcopt = 1500; PBattmax = 3400 W;

### 3.3. Conventional EEMS

The basic purpose of EEMS is to supply the minimum fuel consumption possible while respecting battery and DC bus capacitor power constraints [24]. The fitness function’s objective is to increase the energy supplied by the BS and SC:

(22) $$P_{Batt}^{opt}=\min(\Delta T\cdot P_{Batt}+0.5\cdot C_{bus}\cdot {\left(\Delta V_{bus}\right)}^{2})$$

where *T* represents the sample period, and *V* indicates the charge/discharge voltage. This equation is confirmed by the following optimization:

(23) $$\begin{array}{c} P_{Batt}\leq (SoC-SoC_{\min})V_{Batt}\cdot Q_{Batt}\\ V_{bus}^{\min}-V_{bus}\leq \Delta v\leq V_{bus}^{\max}-V_{bus}\\ P_{Batt}^{charge}\leq P_{Batt}\leq P_{Batt}^{discharge} \end{array}$$

where *V_batt_* and *Q_batt_* are the standard voltage and capacitance of the battery, respectively.

### 3.4. Optimized EMMS-Based WSO

The “fmin” function is a component of the standard EEMS’s programming code [34]. Consequently, a new white shark optimizer is used in place of the function “fmin” to improve the efficiency of EEMS (WSO). The decision factors throughout the optimization procedure are FC power, PFC, battery power, *P_batt_*, and battery SOC.

The lower and upper limits of the decision variables are chosen as indicated in Table 3.

**Figure 9:**
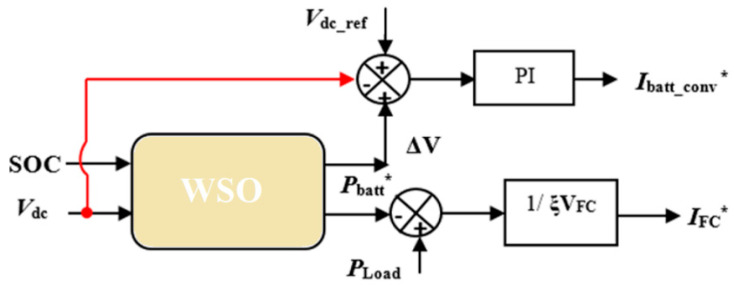
Optimized EEMS-based WSO.

Figure 9 shows the layout of optimized EMMS-based WSO.

WSO is now a revolutionary meta-heuristic optimization tool influenced by great white shark attributes, such as their exceptional hearing and sense of smell when navigating and foraging. The important steps of this algorithm are listed below [35]:

Movement speed towards prey. When a white shark recognizes the location of prey reliant on the waves created by the prey’s activity, it approaches in the following way:

(24) $$s_{i}(t+1)=u\left[s_{i}(t)+\rho_{1}\cdot c_{1}\left(P_{Gbest}(t)-P_{i}(t)\right)+\rho_{2}\cdot c_{2}\left(P_{ibest}(t)-P_{i}(t)\right)\right]$$

where the index *i* (*i* = 1, 2, …, *n*) expresses the white shark order in a population of size *n*, s represents the speed, *P_gbest_* reflects the highest strategic standing vector, *p* is the *ith* white shark’s present position vector, *P_best_* is the current best-achieved position, *c*_1_ and *c*_2_ are two numbers at random from the range [0, 1], *p*_1_, *p*_2_, and *u* are calculated according to the following equations:

(25) $$\rho_{1}=\rho_{\max}+\left(\rho_{\max}-\rho_{\min}\right)e^{-{\left(4t/t_{\max}\right)}^{2}}$$

(26) $$\rho_{2}=\rho_{\max}+\left(\rho_{\max}-\rho_{\min}\right)e^{-{\left(4t/t_{\max}\right)}^{2}}$$

(27) $$u=\frac{2}{\left|2-\tau -\sqrt{\tau^{2}-4\tau }\right|};\;\;\;\tau =4.125$$

Movement towards optimal prey. After they presumably detect the waves caused by the target’s movement or when they see the target’s movements or smell the fragrance of the prey, white sharks continually travel toward their prey. The prey either escapes or leaves its location to search for food. However, its scent in that position is still there. Accordingly, the white shark updates its position as follows:

(28) $$P_{i}(t+1)=\left\{ \begin{array}{l} P_{i}(t)\neg ⊕P_{0}+high\cdot a+low\cdot b;\;rand<m\\ P_{i}(t)+s_{i}(t)/f;\;\;\;rand\geq m \end{array}\right.$$

where “-” is not a sign, *a* and *b* represent one-dimensional binary vectors, *f* is the frequency of the wavy motion, high and low are the upper and lower random search boundaries, and *mv* is described as:

(29) $$m={\left(\left|a_{0}+e^{\frac{t_{\max}/2-t}{a_{1}}}\right|\right)}^{-1}$$

where *a*_0_ and *a*_1_ are constants.

Movement towards the white shark: this phase can be modeled as:

(30) $$m={\left(\left|a_{0}+e^{\frac{t_{\max}/2-t}{a_{1}}}\right|\right)}^{-1}$$

where *a*_0_ and *a*_1_ are constants.

Movement towards the white shark: this phase can be modeled as:

(31) $$P_{i}(t+1)=\left\{ \begin{array}{l} P_{Gbest}(t)+r_{1}\cdot D\cdot \mathrm{sgn}(r_{2}-0.5);\;r_{3}<s_{s}\\ P_{i}(t);\;\;\;otherwise \end{array}\right.$$

wherein *r*_1_, *r*_2_, and *r*_3_ represent the random values ranging in [0, 1], and *D* expresses the distance between the target and the shark.

Fish school behavior: The formula for this phase is provided as follows:

(32) $$P_{i}(t+1)=\frac{P_{i}(t)+P_{i}(t+1)}{2\cdot rand}$$

**Figure 10:**
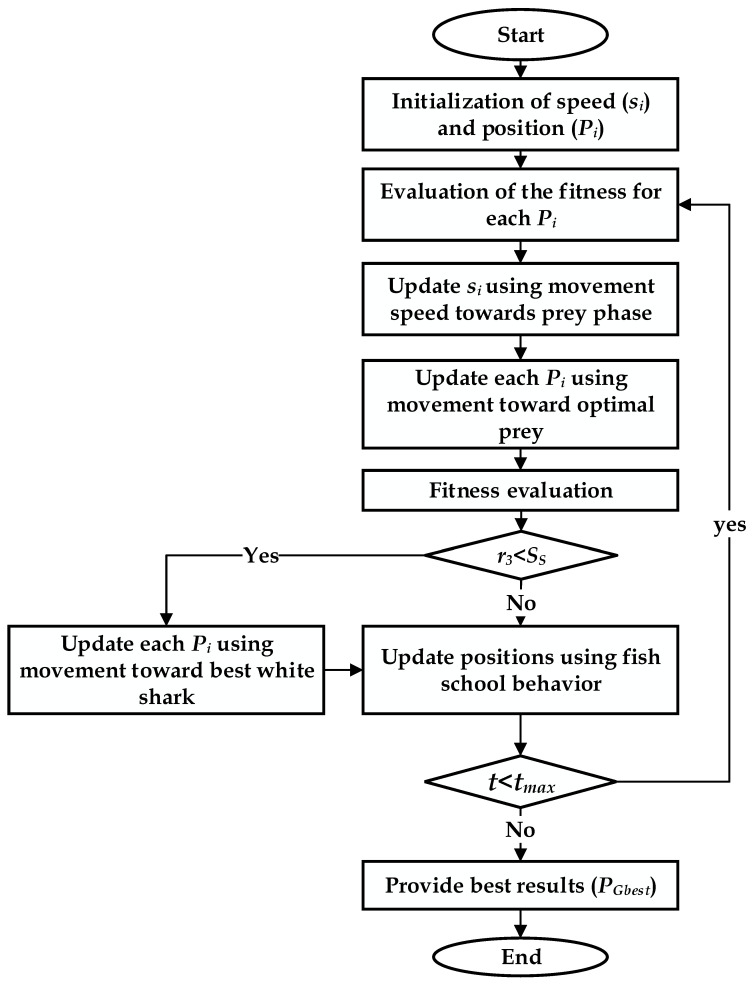
Flowchart for WSO.

The main procedure of WSO is provided in Figure 10.

## 4. Results and Discussion

**Figure 11:**
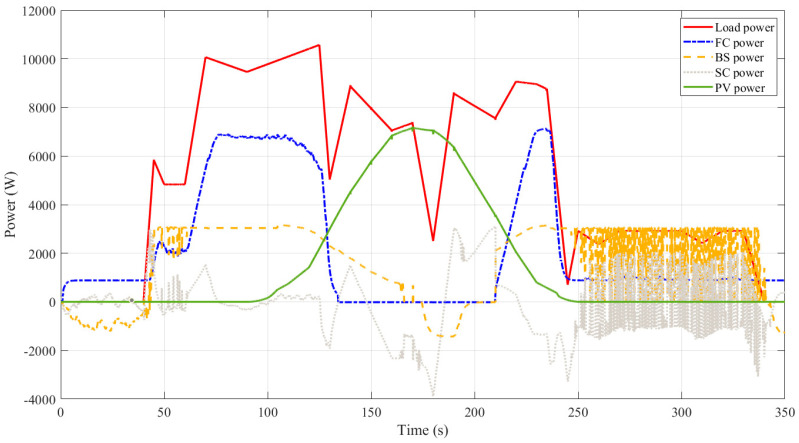
Dynamic produced power by different hybrid system components using WSO.

**Figure 12:**
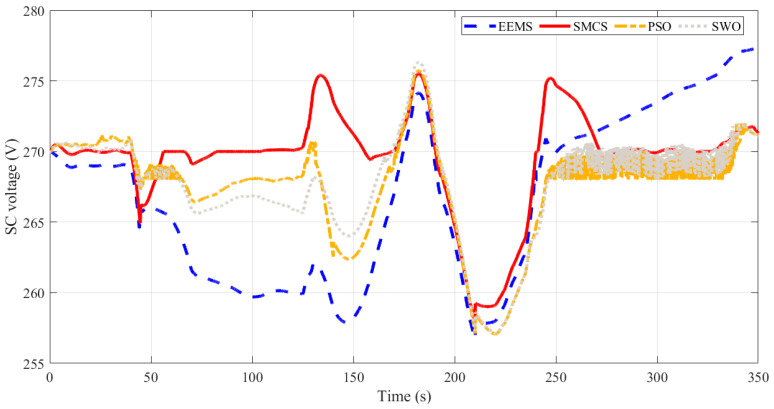
Time response of SC voltage obtained using different EMSs.

The suggested optimized EEMS-based WSO is used to minimize the consumed hydrogen of a hybrid power system shown in Figure 6. The simulation software model’s S-function incorporates the optimized EMMS-based WSO, which is constructed and provided with the following two input variables: the battery SOC and the load profile, whereas the output, the FC and battery banks, are regulated currents. The optimized EMMS-based WSO is compared with conventional EEMS, SMCS, and PSO. Figure 10 demonstrates the load demand that was used (red curve). As presented in Figure 11, at a time of 5 s no load demand is required, and the PEMS starts recharging the BSs with its optimal power (about 1 kW). At 40 s, with no PV power, the extra load demand required is instantly given by the SCs due to its fast dynamics, while the PEMFC power increases gradually. At 45 s, the SCs are discharged below the reference DC link voltage of 270 V, as presented in Figure 12, and the BSs begin delivering power to adjust the DC link voltage back to 270 V. At 48.5 s, the SCs voltage reaches 270 V, and the BSs decreases its power gradually to zero. The PEMFC provides the total demand and continues to recharge the SCs. At 60 s, a fast change in load occurs, and the SCs provide the extra transient demand while the PEMFC power increases gradually. The PV has not yet delivered any power; this changes at *t* = 100 s. Throughout this time, the energy generated by the PV increases to contribute to load-shaving while the excess electricity is used to charge the battery and SC. Likewise, when the PV power has a value, the PEMFC power is zero. At *t* = 250 s, when the battery and SC are simultaneously in the charging mode, the PV is shut off, and the PEMFC returns to share a large amount of load power.

**Figure 13:**
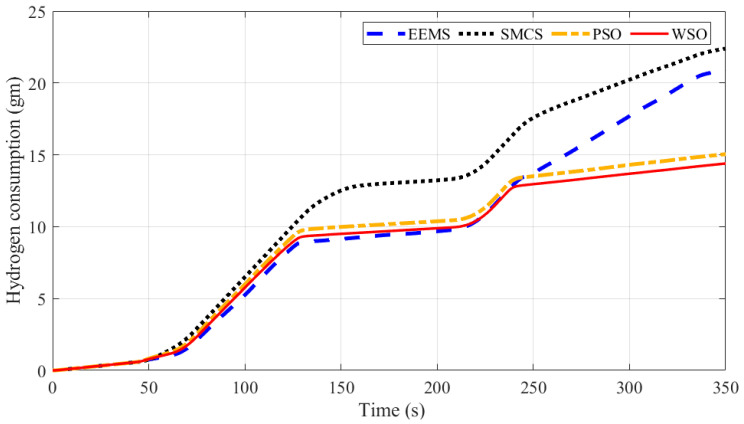
Time response of hydrogen consumption in gm obtained via each optimizer.

Table 2 and Figure 13 present the consumed hydrogen using different EMSs. The results demonstrated the superiority of optimized EEMS-based WSO compared to EEMS, SMCS, and PSO. The EEMS-based WSO reduces hydrogen consumption to a best-obtained value of 14.74 gm, followed by the PSO with 15.05 gm, and the traditional SMCS with a worst-obtained value of 22.39 gm. It is crucial to determine each approach’s efficiency for further research; you may do this by dividing the total power consumed by a PV array, PEMFC, BS, or SC by the total power generated by all of these devices together. The accuracy of each optimization method is shown in Table 4, with the optimized EEMS-based WSO achieving the highest efficiency of 79.24% and the EEMS achieving the lowest efficiency of 72.41%.

**Figure 14:**
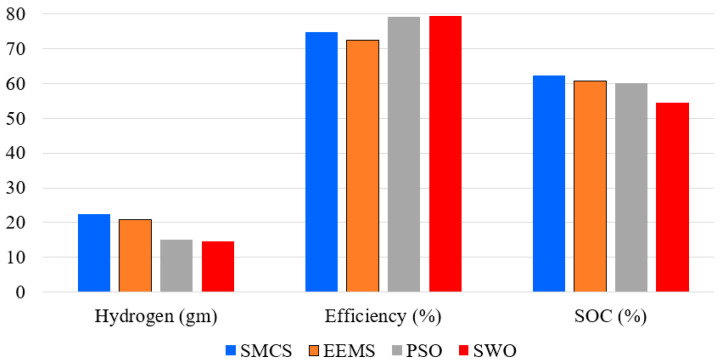
Comparison between different EMSs.

A comparison between different EMSs is presented in Figure 14. Thanks to the proposed EEMS-based WSO, hydrogen consumption has been reduced by 34.17%, 29.47%, and 2.1%, respectively, compared with SMCS, EEMS, and PSO. In addition, the efficiency has been increased by 6.05%, 9.5%, and 0.33%, respectively, compared with SMCS, EEMS, and PSO.

## 5. Conclusions

A new energy management strategy (EMS) using the white shark optimizer technique (WSO) has been proposed to optimally distribute the load demand between the sources in a hybrid PV/PEMFC/BSs/SCs microgrid. The optimized EMS aims to minimize the consumed hydrogen consumption of PEMFC. The optimized EMS avoids the defects of the conventional approach for maximizing external energy (EEMS). The improved EEMS-based WSO is evaluated to EEMS, state machine control (SMCS), and particle swarm optimization (PSO). Two main factors are considered, hydrogen fuel consumption and efficiency. The results reveal that the improved EEMS-based WSO was preferable. Hydrogen consumption has been reduced by 34.17%, 29.47%, and 2.1%, respectively, compared with SMCS, EEMS, and PSO. In addition, the efficiency has been increased by 6.05%, 9.5%, and 0.33%, respectively, compared with SMCS, EEMS, and PSO.6.

## Figures and Tables

**Table 1:** Microgrid benefits and challenges.

	Item	Description
Benefits	Cost optimization	Cost reduction for electricity; Cost savings for fuel and O&M; Independence from changes in energy prices.
	Energy access	Electricity access in isolated places; Support industrial loads; Compatibility with EV charging stations.
	Quality and safety	Backup for power outages; Stabilization of voltage and frequency; Reduced reliance on fuels.
	Environmental and economic benefits	Increasing the usage of renewable energy will minimize the carbon footprint; Rewards, tax advantages, and the avoidance of fines; Reputation.
Challenges	Reliability	High levels of intermittent generation due to the penetration of renewables and random loads.
	Scheduling	Uncertainty of demand and supply.
	Bidirectional power flow	New equipment and protocols are required to consider the bidirectional power flows.
	Plug and play (P&P)	Microgrid-compliant P&P marketing.
	Control	Development and design of control strategies to account for the growth of DGs with power electronics interfaces.
	Grid connection	Resolve grid connection issues such as synchronization.

**Table 2:** The elements of a hybrid system’s electrical characteristics.

PV System		PEMFC	
Type of solar panel	TPB156 × 156-72-P	cells	65
strings	six	Rated voltage	41.15 V
series panels in string	six	Rated current	250 A
Maximum power	295 W	Operating temperature	45 °C
Voltage @ MPP	35.3 V	Efficiency	50%
Current @ MPP	8.36 A		
BSs		SCs	
Rated voltage	48 V	Rated voltage	291.6 V
Capacity	40 Ah	Rated capacitance	15.6 F
Charge rated voltage	55.88 V	Resistance	0.15 Ω
Discharge rated current	17.4 A	Series capacitors	108
Internal resistance	0.012 Ω		

**Table 3:** lower and upper limits.

Parameter	Lower	Upper
PFC (W)	850	8800
Pbatt (W)	1500	3400
SOC	60	90

**Table 4:** Performance of the planned EMS-PPA in comparison to alternative methods.

EMS	Hydrogen (gm)	Efficiency (%)	SOC (%)
SMCS	22.39	74.72	62.21
EEMS	20.9	72.41	60.56
PSO	15.05	78.98	60.1
WSO	14.74	79.24	54.46

## Data Availability

Not applicable.
